# Peripheral Facial Nerve Palsy due to Spontaneous Internal Carotid Artery Dissection

**DOI:** 10.1155/crnm/7669261

**Published:** 2026-01-30

**Authors:** Benjamin Dejakum, Michael Knoflach, Stefan Kiechl, Lukas Mayer-Suess

**Affiliations:** ^1^ Department of Neurology, Medical University of Innsbruck, Anichstrasse 35, Innsbruck, 6020, Austria, i-med.ac.at

## Abstract

A man in his 50s experienced novel, continuous, and progressive headache and neck pain prior to the onset of left‐sided peripheral facial nerve palsy. Sequential palsies of left lower Cranial Nerves IX and XII followed. Imaging showed spontaneous cervical artery dissection (sCeAD) of the ipsilateral internal carotid artery. Lower cranial nerve palsies in sCeAD are a frequent result of a local mass effect exerted by the formation of a mural hematoma. The only close topographical relationship between the facial nerve and the internal carotid artery is within the petrous part of the temporal bone but still separated in two different bony canals (facial canal and carotid canal). Thus, a mural hematoma of an internal carotid artery dissection could not cause compression of the facial nerve. In the rare case of facial nerve palsy due to sCeAD, hypoperfusion of the vasa nervorum is the most likely cause. As sCeAD is one of the main reasons for stroke in the youth, it is critical to know and identify potential red flags in patients with peripheral facial nerve palsy, which should lead to additional vascular imaging.

## 1. Background

This case highlights peripheral facial nerve palsy resulting from hypoperfusion of the vasa nervorum in the context of sCeAD. sCeAD is one of the main causes of ischemic stroke in the youth. Local symptoms caused by sCeAD, such as cranial nerve (CN) palsies and local pain, often predate ischemic events. It is crucial to identify red flags in patients with peripheral facial nerve palsy or multiple CN palsies, such as Horner’s syndrome, acute onset of unknown progressive head pain and/or neck pain, which typically does not respond well to analgesics, which should prompt the clinicians to additional diagnostic workup including vascular imaging.

## 2. Case Presentation

A man in his 50s woke up with left‐sided, occipital‐predominant headache, and neck pain of mild intensity (Visual Analog Scale [VAS] 3 of 10) different to his known migraine headache (now pulling, otherwise throbbing). The pain was progressive and did not respond to his usual analgesics. Five days later, he experienced left‐sided facial paralysis. Patient history was uneventful, with no recent trauma or infection reported. Neurologic examination revealed isolated left peripheral facial nerve (VIIth nerve) palsy; however, in the context of novel progressive head/neck pain, he was admitted for further evaluation. Within the following days, sequential palsies of peripheral caudal CNs occurred, first left‐sided palatal palsy (IXth nerve) followed by tongue deviation to the left (XIIth nerve). Initial diagnostic workup showed an atypical conduction block in VIIth nerve motor–evoked potentials, normal cerebrospinal fluid parameters (including glucose, protein, erythrocytes, leukocytes and cytology) and no pathologic findings in the blood sampling. Magnetic resonance imaging (MRI) of the head, neck, and abdomen revealed no cerebral ischemic lesion, trauma, or malignancy, but a T1‐fat‐saturated hyperintensity along the entire length of the left internal carotid artery (ICA) up to the cavernous part causing vessel occlusion, consistent with sCeAD of the ICA. An ultrasound of extra‐ and intracranial vessels confirmed the left‐sided ICA occlusion with insufficient but intact intracranial collateralization (marked side‐by‐side difference in middle cerebral artery perfusion) through anterior cross‐flow and flow reversal of the left‐sided ophthalmic artery (Figure 1).

Figure 1(a) Occlusion of the left internal carotid artery (b) due to spontaneous cervical artery dissection (sCeAD)–related vessel wall hematoma in T1‐fat‐saturated MRI and (c) retrograde perfusion of the left‐sided ophthalmic artery.

**Figure:**
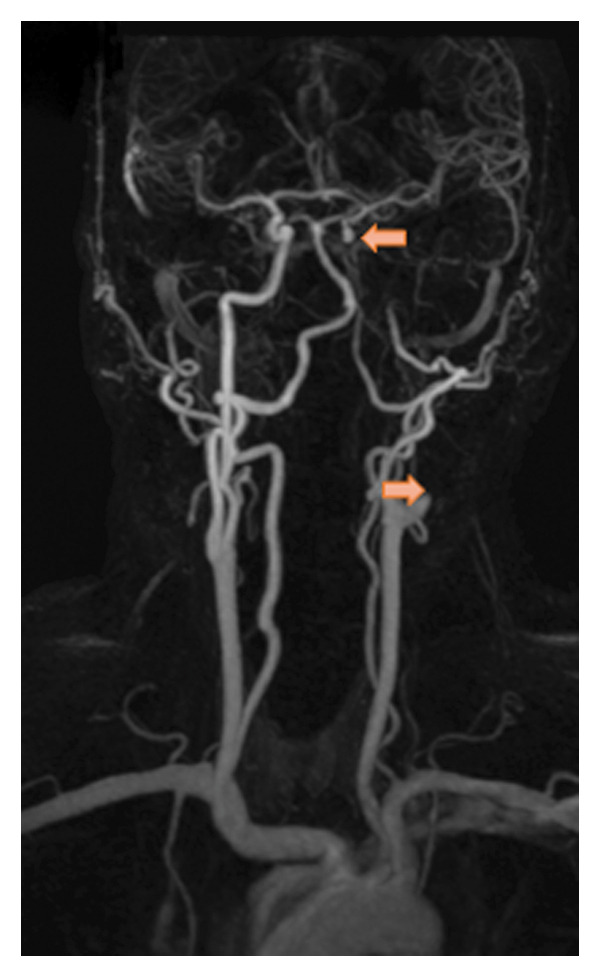
(a)

**Figure:**
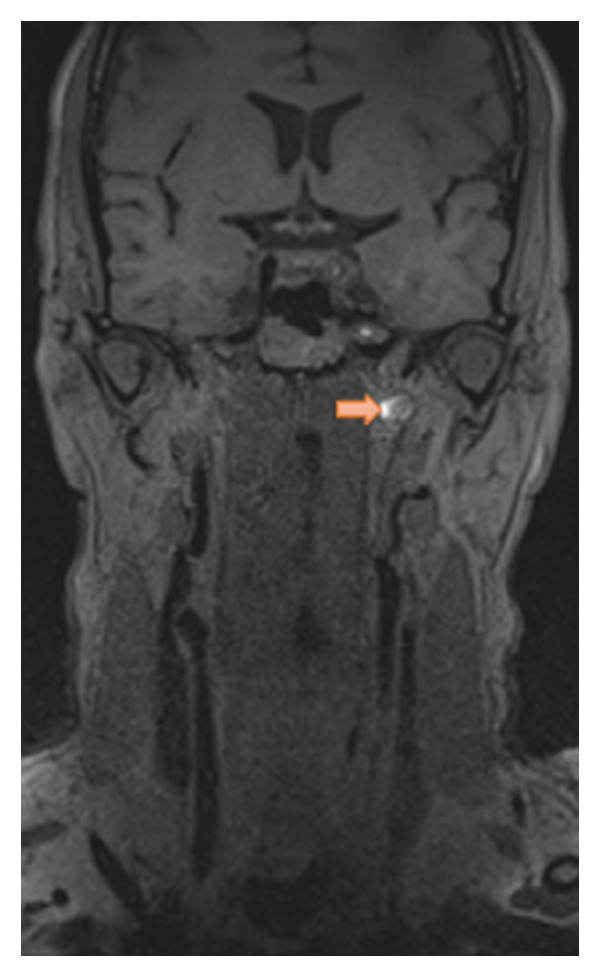
(b)

**Figure:**
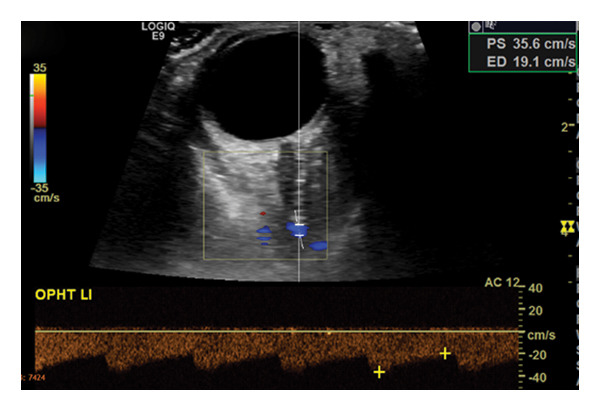
(c)

The patient was treated with 100 mg of acetylsalicylic acid once a day. At a 3‐month follow‐up, the patient reported that all symptoms resolved 3 weeks after onset. Follow‐up MRI of the head and neck revealed residual occlusion of the left ICA, but a prominent left‐sided ophthalmic artery was consistent with potent collateralization. The patient returned to work and was independent in all his daily activities. At the 12‐month follow‐up, the patient reported no further symptoms, and the dissected ICA showed signs of recanalization.

## 3. Discussion

CN palsies in sCeAD are usually confined to caudal ones with XIIth nerve being most commonly affected [1]. The cause for lower CN palsies, as in our case (IX and XII), is the anatomical vicinity of these nerves to the ICA right below the skull base. IXth, Xth, and XIth nerves leave the skull collectively through the jugular foramen, and XIIth nerve through the hypoglossal canal. Close to these gates of exit, the ICA enters the skull through the carotid canal together with the carotid plexus (Figure 2).

FIGURE 2(a) Drawing of an anatomical axial section through the neck from Sturzenegger M. et al. at the level of the first cervical vertebral body (atlas). The close relationship between the ICA and the IXth, Xth, XIth, and XIIth cranial nerves is highlighted in the magnified cutting. (1) Nasal cavity; (2) maxillary sinus; (3) facial muscles; (4) jaw muscles; (5) neck muscles; (6) atlas body; (7) parotid gland; (8) parapharyngeal space [2]. (b) Schematic drawing of the carotid sheath; (1) internal carotid artery; (2) internal jugular vein; (IX) glossopharyngeal nerve; (X) vagus nerve; (XI) accessory nerve; (XII) hypoglossal nerve.

**Figure:**
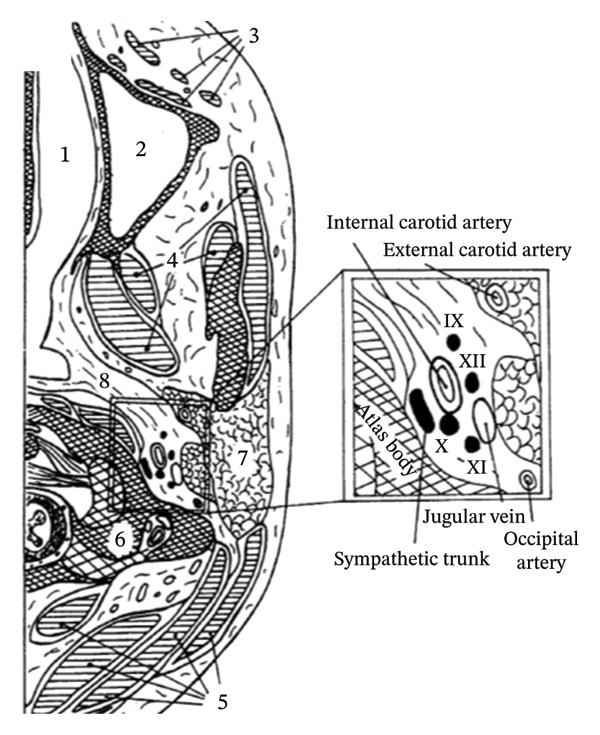
(a)

**Figure:**
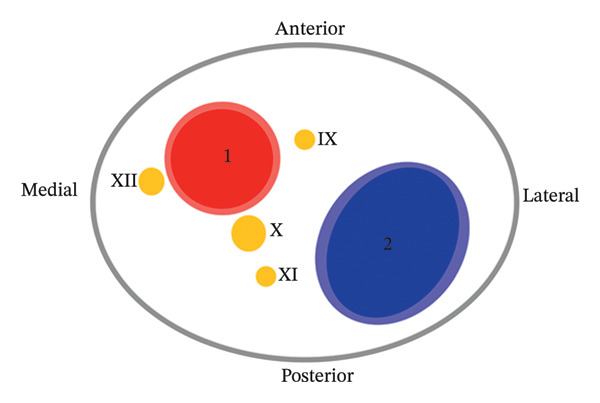
(b)

ICA dissection–associated vessel wall hematoma can subsequently exert local mass effect to adjacent structures, causing CN palsies [3]. sCeAD causing peripheral VIIth nerve palsy is rare, and local mass effect is an unlikely reason due to its anatomical course. Only seven other case reports exist in the literature, two of which are due to traumatic cervical artery dissection (Table 1).

**TABLE 1:** Literature review of patients with peripheral VIIth palsy due to CeAD.

Age/sex	VII side	Dissection and status	Cause of CeAD	Other symptoms	Initial symptom	Other cranial nerves	Ischemia	Recovery	Ref
42/m	Right‐sided	Right ICA (status: N/A)	Traumatic	Right‐sided head/neck pain, Horner’s syndrome	N/A	None	None	Duration N/A: incomplete	Fioravanti et al. [4]
44/m	Left‐sided	Left ICA (status: occlusion)	Spontaneous	Partial Horner’s syndrome left, left‐sided head/neck pain, left hyperacusis	Head/neck pain	None	N/A	After 6 months: resolved	McCarron et al. [5]
49/m	Left‐sided	Bilateral ICA (status: occlusion)	Spontaneous	Left‐sided head/neck pain, left partial Horner’s syndrome	Head/neck pain	None	None	After 2 months: incomplete	Gout et al. [6]
53/m	Left‐sided	Left ICA (status: high‐grade stenosis)	Spontaneous	Left‐sided head/neck pain, dysphagia, left‐sided palatal palsy, tongue deviation to the left	Head/neck pain	Left IX, X, XII	None	After 6 months: resolved	Panisset et al. [7]
55/m	Right‐sided	Right ICA (status N/A)	Spontaneous	Head/neck pain, dysphagia, tongue deviation to right side, right partial Horner’s syndrome	Head/neck pain	Right X, XII	None	After 6 weeks: incomplete	Majeed et al. [8]
17/w	Left‐sided	Left ICA (status: oclussion)	Traumatic	Deviation of the tongue to the left	Left VII	Left XII	None	N/A	Naik et al. [9]
44/m	Right‐sided	Bilateral VAs/ICAs (left ICA status: high‐grade stenosis)	Spontaneous	Severe head/neck pain, left Horner’s syndrome	Head/neck pain	None	Left PICA	After 2 months: incomplete	Chung et al. [10]
52/m	Left‐sided	Left ICA (status: occlusion)	Spontaneous	Left partial Horner’s syndrome, tongue deviation to the left, left‐sided palatal palsy	Head/neck pain	Left IX, XII	None	After 3 weeks: resolved	Current case

*Note:* Literature review of patients with peripheral VIIth palsy due to CeAD. VII: facial nerve; CeAD: cervical artery dissection; IX: glossopharyngeal nerve; X: vagus nerve; XII: hypoglossal nerve.

Abbreviations: ICA, internal carotid artery; PICA, posterior inferior cerebellar artery; VA, vertebral artery.

The most probable mechanism for sCeAD‐related VIIth nerve palsy is hypoperfusion of the vasa nervorum, which correlates to all reported cases having high‐grade stenosis or occlusion of the ICA (Table 1) [2]. The arterial supply of VIIth nerve stems from three main sources are as follows: (1) labyrinthine artery (origin: anterior inferior cerebellar artery), (2) stylomastoid artery (origin: external carotid artery [ECA]), and (3) superficial petrosal artery (origin: middle meningeal artery [MMA]—branch from the ECA) [11]. However, MMA may originate from the ICA as an anatomical variant [2]. Irrespective of MMAs origin, ICA–sCeAD with high‐grade stenosis or occlusion may lead to (1) direct hypoperfusion of the MMA, if it branches from the ICA, or (2) a steal phenomenon from the MMA, if it originates from the ECA due to the need of intracranial collateralization through ophthalmic artery in ICA occlusions.

## 4. Conclusion

As it relates to our case, MRI revealed a prominent MMA originating from the ECA. All symptoms resolved, including VIIth nerve palsy, after the initially insufficient collateralization stabilized within 3 weeks, assuming that a steal phenomenon is more probable in this patient. Concerning clinical identification of patients at risk of sCeAD‐related VIIth nerve palsy, we described a specific type of head and neck pain associated to sCeAD, which fits this case (acute onset, pulling with mild‐to‐moderate intensity, progressive, and not responsive to oral analgesia) and 6 of the 7 reported cases in the literature (Table 1) [12].

### 4.1. Key Takeaways

- Local symptoms, such as head/neck pain, Horner’s syndrome, pulsatile tinnitus, and CN palsies are the most frequent symptoms attributable to sCeAD [1].
- CN palsies in sCeAD are usually confined to caudal ones (most commonly XIIth nerve palsy).
- Peripheral VIIth nerve palsy due to sCeAD is rare and most likely stems from hypoperfusion of the vasa nervorum.
- A sCeAD‐specific type of head and/or neck pain, such as acute onset, pulling with mild‐to‐moderate intensity, progressive, not responsive to oral analgesia, and can assist clinicians in identifying VIIth nerve palsy patients with underlying sCeAD [12].

## Funding

Open access funding was provided by Medizinische Universitat Innsbruck/KEMÖ

## Ethics Statement

This study involves human participants but an ethics committee or institutional board exempted this study. For this case report, no approval by an ethics committee was needed.

## Consent

All the patients allowed personal data processing, and informed consent was obtained from all individual participants included in the study.

## Conflicts of Interest

The authors declare no conflicts of interest.

## Data Availability

The data that support the findings of this study are available on request from the corresponding author. The data are not publicly available due to privacy or ethical restrictions.
